# Clinical and Virological Aspects of HBV Reactivation: A Focus on Acute Liver Failure

**DOI:** 10.3390/v11090863

**Published:** 2019-09-16

**Authors:** Olympia E. Anastasiou, Martin Theissen, Jens Verheyen, Barbara Bleekmann, Heiner Wedemeyer, Marek Widera, Sandra Ciesek

**Affiliations:** 1 Institute of Virology, University Hospital Essen, University of Duisburg-Essen, 45147 Essen, Germany; Jens.Verheyen@uni-due.de (J.V.); Barbara.Bleekmann@uk-essen.de (B.B.); marek.widera@uni-due.de (M.W.); Sandra.Ciesek@kgu.de (S.C.); 2 Department of Bioinformatics and Computational Biophysics, University of Duisburg-Essen, 45117 Essen, Germany; Martin.Theissen@uni-due.de; 3 Department of Gastroenterology and Hepatology, University Hospital Essen, 45147 Essen, Germany; Heiner.Wedemeyer@uk-essen.de; 4 German Center for Infection Research, DZIF, 38124 Braunschweig, Germany; 5 Institute of Medical Virology, University Hospital Frankfurt, 60596 Frankfurt am Main, Germany

**Keywords:** hepatitis B, HBV, reactivation, NGS, ALF, acute liver failure

## Abstract

Hepatitis B virus (HBV) reactivation in immunosuppressed patients can cause considerable morbidity and mortality. The aim of our study was to evaluate factors associated with acute liver failure (ALF) in HBV reactivation. Clinical, laboratory, and virological data of 87 patients with HBV reactivation were analyzed retrospectively. Teno torque virus (TTV) plasma loads were measured as a measure of immune competence. HBV genomes isolated from 47 patients were analyzed by next-generation sequencing. A functional analysis of identified HBsAg mutants was performed. In patients with ALF the diagnosis was significantly later confirmed than in the non-ALF group. Patients diagnosed during immunosuppression had a milder clinical course compared to later diagnosed patients (*p* = 0.018, *OR* = 4.17). TTV viral loads did not differ significantly between the two groups. The HBV genomes isolated from ALF patients had higher viral complexity. A mutation in C-region of HBsAg (L216*), was associated with reduced HBsAg production and secretion. Patients diagnosed with HBV reactivation during immunosuppression had a milder clinical course compared to patients diagnosed during immune reconstitution. ALF was associated with higher viral complexity. An HBsAg mutation (L216*) was found to be more frequent in ALF patients and was associated with reduced HBsAg production and secretion.

## 1. Introduction

Hepatitis B virus (HBV) infection remains a major global health issue affecting millions of people worldwide and is a leading cause of morbidity and mortality. After infection, the virus persists in the liver, not only in patients developing chronic infection, but also in patients with serological recovery from the infection (HBsAg negative, anti-HBc positive with or without anti-HBs) [1]. HBV reactivation has been defined as the reappearance or rise of HBV DNA in patients with inactive or resolved HBV infection. Although it can occur spontaneously, it is mostly triggered by immunosuppression, often due to chemotherapy (for example treatment with rituximab), allogeneic hematopoietic stem cell transplantation (aHSCT) or solid organ transplantation [2,3].

The clinical manifestation of HBV reactivation ranges from asymptomatic to acute liver failure (ALF). Its outcome may be immunological control or persistence of HBV infection [2,4]. HBV reactivation can be divided into three phases as described by Hoofnagle [5]: Firstly, the reappearance of HBsAg or HBV DNA in the serum of previously HBsAg-negative patients or an increase in HBV DNA serum levels in HBsAg-positive patients can be observed. During the second phase, HBV DNA levels increase accompanied by an increase of liver transaminases with varying degrees of hepatic injury. In the third phase, the hepatic injury resolves spontaneously or as a result either of withholding immunosuppressive therapy or under antiviral treatment.

The outcome of patients with HBV reactivation varies and immunosuppression can have both positive and negative aspects. On the one hand, HBV reactivation triggers an unchecked viral replication, on the other hand hepatic injury is often milder, as attested by low transaminase levels [2,5]. Immunosuppression is, however, a vague term and it is often difficult to assess its degree in patients under immunosuppressive treatment. The presence of circular ssDNA genome of the Teno torque virus (TTV), which is by itself asymptomatic and not associated with clinical diseases, has been proposed as a novel surrogate biomarker for the efficacy of immunosuppression in different patient cohorts and is even used to guide the level of immunosuppression in lung transplant recipients [6,7].

Another aspect of HBV reactivation in immunosuppressed patients is that chemotherapeutic or immunosuppressive drugs themselves can lead to hepatic injury. This can be due to chemotherapy-induced hepatotoxicity, the toxicity of other drugs given during or after the treatment (for example antifungal agents), or exacerbation of pre-existing liver disease, as in the case of HBV reactivation [8,9,10]. In a retrospective multicenter study, including 156 patients with HBV-induced acute liver failure (ALF), 18% of all ALF patients received immunosuppressive therapy. Their chance of survival was found to be diminished compared to patients not receiving immunosuppression [11].

Previous studies have demonstrated that viral factors, such as increased variability in HBV core, preS2 region, and HBsAg, as well as host factors (e.g., the presence of certain human leukocyte antigen (HLA) class II locus alleles), are associated with the development of ALF in the case of an acute HBV infection [12,13,14]. Furthermore, HBV reactivation has been shown to correlate with HBsAg mutations associated with enhanced capability to evade immune response, some of them were only present as minority variants as shown per next-generation sequencing (NGS) [15].

The aim of our study was to investigate viral and host factors, which may contribute to ALF development in patients with HBV reactivation. To this purpose, we evaluated the impact of clinical and virological parameters and the degree of immunosuppression on the outcome of patients with HBV reactivation. Furthermore, we investigated the extent of viral genetic diversity, heterogeneity, and the existence of specific mutations associated with worse outcomes. 

## 2. Materials and Methods

### 2.1. Study Design

A retrospective analysis of 87 patients diagnosed with HBV reactivation at the University Hospital Essen, Germany between 2000 and 2019 was performed. Enrolled patients fulfilled the criteria for HBV reactivation proposed by Hwang et al. and had a reappearance of serum HBV DNA or a marked rise in serum HBV DNA (>2 log IU/mL from baseline level) during or after the administration of immunosuppressive therapy or due to hematological malignancy [2]. A total of 16 of 86 patients fulfilled all criteria for ALF: a) hepatic encephalopathy of any degree, b) evidence of coagulopathy with international normalized ratio (INR) of ≥1.5, c) acute illness onset <26 weeks, and d) no evidence of cirrhosis [16]. Patients with HBV/HDV coinfection or HIV infection were excluded from the study.

The outcome was defined by the clinical status six months after the initial diagnosis of HBV reactivation and was classified as either recovery with functional HBV cure (HBsAg/HBV DNA undetectable), chronic hepatitis B (continued HBV replication/presence of HBsAg) or non-recovery with liver transplantation and/or death. Six patients were lost to follow up. Abdominal ultrasonography was performed in 68 patients at the time point of diagnosis of HBV reactivation. Hepatic steatosis was diagnosed in cases with increased liver/kidney echogenicity and posterior attenuation.

This retrospective study was carried out in accordance with the Declaration of Helsinki and the guidelines of the International Conference for Harmonization for Good Clinical Practice. The study was approved by the local Ethics Committee at the University Hospital Essen (Institutional Review Board, 2015, 15-6543-BO). Informed consent was waived due to the retrospective character of the study.

### 2.2. Laboratory Parameters

Routine laboratory parameters such as liver transaminases and coagulation parameters were measured in the central laboratory of the University Hospital Essen and taken from patient charts. Serological parameters were measured in the Institute for Virology; HBsAg, anti-HBs, anti-HBe, HBeAg, and anti-HBc (total, IgM, and IgG) were measured with Architect Abbott i2000SR according to the manufacturer’s instructions (Abbott, Wiesbaden, Germany). HBV viral load was quantified by real-time PCR performed on real-time Abbott (m2000) and Siemens bDNA HBV test platform according to the manufacturer’s instructions. TTV viral loads were determined by an in house real-time PCR with valid ring test certificate (QCMD). The assay was performed on a Rotor-Gene Q instrument (Qiagen, Hilden, Germany) using a standard curve TTV genotype 1a DNA (AB017610.1) cloned in a pCR2.1 vector as described previously [6]. 

### 2.3. Sequencing of HBV Genome

Archived plasma/serum samples obtained for diagnostic purposes were retrospectively used for DNA extraction. DNA extraction was performed with QIAamp DNA mini kit (Qiagen). PCR for the whole HBV genome was performed as previously described [12]. For genotype assessment, detection of immune escape mutations and stop codon mutations in HBsAg the geno2pheno platform was used [17]. Partial or full HBV genome sequencing was performed by NGS on a MiSeq platform (Illumina) and was possible for 47 patients. The preS1, preS2, small hepatitis B surface protein (SHB), reverse transcriptase (RT), precore, core, and HBx protein regions were compared to genotype-specific reference sequences of these regions, taken from the dataset of geno2pheno for SHB and RT and from HBVdb for all other regions [18]. For the above-mentioned comparison, we used sequences present in ≥10% of the reads. 

A comparison was carried out only for samples with coverage >100× over the complete reference region of interest (core, precore, HBsAg, preS1, preS2, und HBx). Positions with at least one non-reference amino acid present in >10% of the quasispecies were considered as mismatches. The genetic complexity of the viral quasispecies was evaluated for each viral region by calculating the position-wise Shannon entropy at the nucleotide level. The mean viral complexity in each sample and region was determined by averaging over the entropy, accounting only for positions with coverage >100× [19]. 

### 2.4. Construction of HBsAg Mutants and Transient Transfection

A stop codon mutation was found more frequently in the ALF group (SHB L216*, see Table 3) and was selected for further analysis. In addition, two more variants were used as controls, the SHB D144E which could be found in both groups and the SHB W172*, and its impact on HBsAg secretion has been already described before [20]. In order to investigate the impact of these mutations on HBsAg recognition and quantification, a plasmid encoding the HBsAg linked to a streptavidin tag (strep-tag) was used to transfect Huh7 cells. The amount of strep-tagged HBsAg released in culture supernatants was then quantified using different enzyme-linked immunosorbent assays (ELISAs): a specifically designed ELISA capable of recognizing the Strep-tag linked to the HBsAg (defined as Strep-tag ELISA, see below) and an ELISAs directed against the HBsAg protein (Architect, Abbott, IL, USA). 

In detail, a Strep-tag HBsAg genotype D was cloned in the pEXPR-IBA 44 (iba-lifesciences) Vector and was subsequently used as a template to generate the HBsAg mutants, D144E, W172* and L216*. Mutants were generated via site-directed mutagenesis. Detailed site-directed mutagenesis strategies are available upon request. Huh7 cells were grown in a 37 °C humidified atmosphere containing 5% CO_2_, using Dulbecco’s modified Eagle’s medium (Life Technologies, Inc., Gaithersburg, MD, USA) supplemented with 10% fetal bovine heat-inactivated serum and with 100 U/mL penicillin + 100 μg/mL streptomycin + 2 mM l-glutamine + nonessential amino acid solution. The cells were transiently transfected with the pEXPR-HBsAg (wild type (wt) or mutant) by using TransIT-2020 Transfection Reagent (Mirus Bio LLC, Madison, WI, USA) according to the manufacturer’s instructions. Seventy-two hours after transfection, supernatants and cell lysates were collected, clarified by centrifugation at 3000× *g* for 5 min, and stored at −20 °C. Four transfection experiments were performed. Transfection efficiency was monitored by co-transfection of a vector expressing luciferase (pTA-Luc) and measured with a luciferase assay.

### 2.5. Quantification of HBsAg and Strep-Tag ELISA

HBsAg concentration in the cell lysates and supernatants was measured using Architect Abbott according to the manufacturer’s instructions (Abbott, Germany) and an enzyme-linked immunosorbent assay (ELISA) targeting the Strep-tag. StrepMAB-Immo coated microplates (IBA) were used as immobilization device. Cell supernatants and lysates were diluted with Tris-buffered saline 0.1% Tween-20 (TBST, pH 7.4, 0.5% BSA) in a ratio of 1:1. Samples were prepared in duplicate at 100 µL/well. Upon addition of samples, the plate was incubated for 1 h at room temperature under constant agitation. Wells were washed 5× with TBST 0.5% BSA. In the next step, 100 µL of HRP conjugated StrepMAB-Classic (IBA; 1:15.000) was added and the plate was incubated for 1 h at RT. The wells were again washed 5× with TBST 0.5% BSA followed by the addition of 100 µL 3,3′,5,5′-tetramethylbenzidine substrate (1-Step Ultra TMB-ELISA, Thermo Fisher, Germany). To cease the reaction 50 µL stop solution (2 M H_2_SO_4_) was added. Absorbance was measured at 450 nm with a microplate reader.

### 2.6. Statistical Analysis

Categorical and continuous data were compared between the patient groups. Statistical significance was assessed by Chi-Square or Fisher’s exact test for categorical data. Continuous data were expressed as median (interquartile range). Unpaired *t*-Test, Mann–Whitney *U* and Kruskal Wallis tests were used to compare continuous variables. Two-tailed *P* values less than 0.05 were considered statistically significant. Statistical analysis was performed using SPSS software (v21, SPSS Inc., Chicago, IL, USA), GraphPad Prism 6.0 (GraphPad, CA, USA), R software and the platform VassarStats (http://vassarstats.net).

## 3. Results

**Figure 1:**
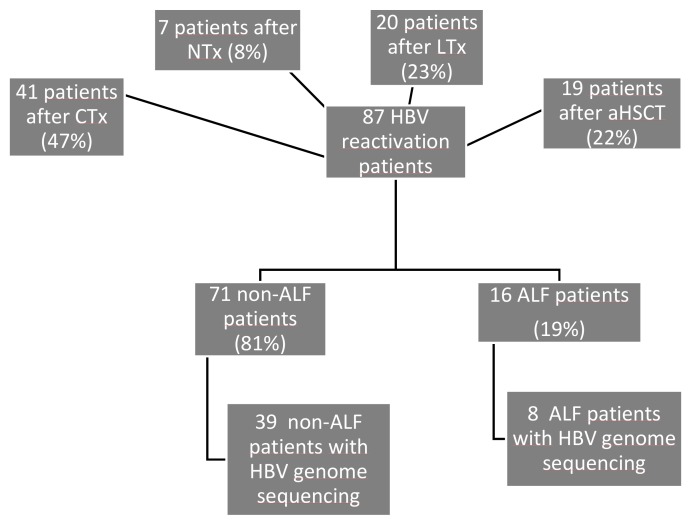
Flowchart of the patients analyzed in this study. ALF: acute liver failure, CTx: group of patients with hematological or oncological diseases after chemotherapy, aHSCT: group of patients after allogeneic hematopoietic stem cell transplantation, NTx: group of patients after kidney transplantation, LTx: group of patients after liver transplantation.

We identified 87 patients with HBV reactivation. 41 patients (47%) with HBV reactivation received chemotherapy for hematological, oncological, or non-malign diseases or had a hematological disease (five patients with chronic lymphocytic leukemia without immunosuppressive treatment, all other patients during or after chemotherapy), 19 patients (22%) underwent allogeneic bone marrow transplantation, 7 patients (8%) underwent kidney transplantation, and 20 patients (23%) were liver transplanted (Figure 1). Five out of 87 patients (6%) developed HBV reactivation under antiviral prophylaxis (Table 1). As shown in Figure 1 16 out of 87 (19%) patients developed ALF. In the ALF group we observed resolution of HBV infection in 4 patients (25%), 1 patient (6%) developed chronic hepatitis B virus infection, while 11 patients (69%) died and/or underwent liver transplantation. In the non-ALF group we observed resolution of HBV infection in 20 patients (28%), while 36 patients (51%) developed chronic hepatitis B virus infection and 9 patients died (13%). Six patients of the non-ALF group (8%) were lost to follow up. All ALF patients received antiviral treatment after HBV reactivation, while almost a quarter of the non-ALF patients did not.

### 3.1. Clinical Parameters Associated with ALF in Patients with HBV Reactivation

Next, we evaluated the clinical and demographical parameters of our cohort. An overview of all data is shown in Table 1. Age, sex, body mass index, and presence of hepatic steatosis had no significant impact on the course of HBV reactivation. As expected, patients with ALF had a worse outcome and reduced liver function as well as higher liver transaminases in comparison to patients without ALF. Interestingly, patients with HBV reactivation and hematological or oncological malignancies receiving chemotherapy had an increased risk of developing ALF compared to all other patients (*p* = 0.024, *OR* = 4.34). The time point of HBV reactivation diagnosis differed affected to outcome of the patients: Patients diagnosed during immunosuppression (during chemotherapy and all transplanted patients) had a milder clinical course compared to patients diagnosed later (*p* = 0.0.18, *OR* = 4.25 as shown in Table 1. Five patients developed HBV reactivation under antiviral prophylaxis, three after liver transplantation and two after aHSCT. All patients had low HBV viral loads and none developed ALF. Sequencing of the RT region was performed in one case, where no drug resistance associated mutations were to be found. The therapy with entecavir was continued and the patient was lost to follow up. In one patient, HBV DNA became undetectable without switching therapy. In the last three patients, therapy was switched to tenofovir. In two of them, HBV DNA became undetectable later, the last patient was lost on follow up. Seventeen patients did not receive antiviral treatment after diagnosis of HBV reactivation. The thought process leading to this clinical decision is not always documented. None of them developed liver failure, most (*n* = 9) had low viral loads and HBV infection resolved spontaneously in seven of them. One patient died 3 days after diagnosis due to a non HBV associated cause and another patient was non-adherent.

### 3.2. Virological Parameters Associated with ALF in Patients with HBV Reactivation 

HBsAg positive patients with low replicative baseline HBV infection had a greater risk to develop ALF compared to HBsAg negative patients (*p* = 0.003, *OR* = 8.17) and to patients receiving a liver transplant from an anti-HBc+ donor (*p* = 0.017, *OR* = 9.33). We could not detect significant differences in HBV viral load and signal to cutoff (S/Co) for anti-HBc IgG and IgM as well as HBV genotype in the two groups at the time of reactivation. Furthermore, patients with high titer of HBsAg (*p* = 0.012) and detectable anti-HBe antibodies at reactivation had a higher risk to develop ALF (*p* = 0.007, *OR* = 5.5) as shown in Table 2. TTV viral load as a marker for immunosuppression did not differ significantly between patients with ALF versus non-ALF. In addition, there was no difference in TTV viral load in patients diagnosed during immunosuppression (*n* = 45, 24,600 (605,568)) and patients diagnosed after immunosuppression (*n* = 27, 63,500 (874,365)) (*p* = 0.740), nor in patients achieving resolution of the HBV infection (*n* = 19, 8200 (401,703)) compared to patients developing chronic hepatitis B (*n* = 31, 61000 (564,355)) (*p* = 0.727). All parameters apart from the baseline status were measured at the time point of HBV reactivation diagnosis.

### 3.3. Viral Complexity of the HBV Quasispecies of HBV Reactivation Patients According to the Presence of Liver Failure

**Figure 2:**
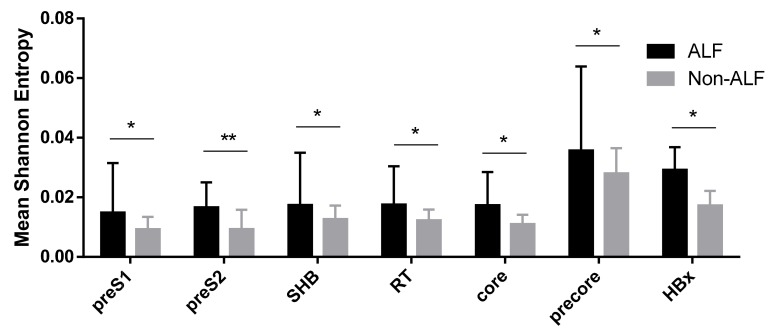
The small hepatitis B surface protein (SHB), preS1, preS2, reverse transcriptase (RT), precore, core, and HBx regions were next-generation sequencing (NGS) sequenced. The mean Shannon entropy values were calculated for each viral genomic region for patients with HBV reactivation with and without liver failure. Results are presented as median (IQR). Patients with liver failure had consistently higher values in all evaluated regions (preS1, preS2, SHB, RT, precore, core, and HBx). The comparison between the two groups was performed using the Mann–Whitney *U* test. SHB = small hepatitis B surface protein, RT = reverse transcriptase, * = *p* < 0.05, ** = *p* < 0.01.

The viral complexity of the HBV quasispecies of our patient cohort was evaluated by Shannon entropy. Patients with ALF had higher mean entropy than non-ALF patients in the preS1 region (0.015 (0.021) vs. 0.009 (0.006), *p* = 0.015). As shown in Figure 2, a comparable trend was observed for the preS2 region (0.017 (0.012) vs. 0.011 (0.006), *p* = 0.01), the SHB region (0.017 (0.021) vs. 0.013 (0.006), *p* = 0.036), the RT region (0.018 (0.016) vs. 0.012 (0.005), *p* = 0.016), the core region (0.017 (0.016) vs. 0.011 (0.005), *p* = 0.032), the precore region (0.036 (0.033) vs. 0.028 (0.014), *p* = 0.012), and the HBx region (0.029 (0.015) vs. 0.017 (0.008), *p* = 0.01).

### 3.4. Variability in SHB, PreS1, PreS2, Core, Precore, and HBx Compared to Genotype A Specific Reference Sequence

**Figure 3:**
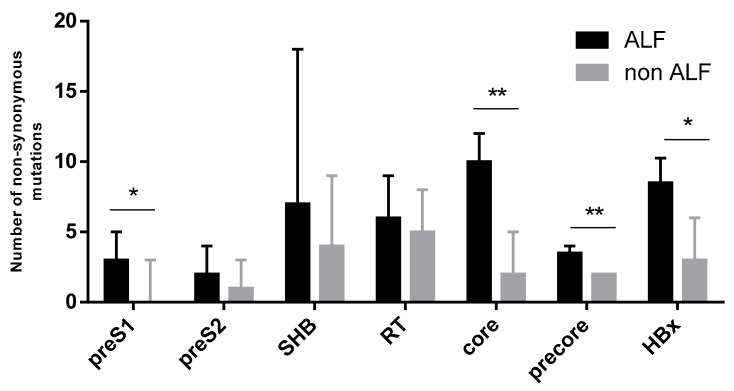
The SHB, preS1, preS2, RT, precore, core, and HBx regions were NGS sequenced. Results are presented as the medians (interquartile range) of the number of nonsynonymous mutations in each region of the patient sequences when compared with a genotype-specific reference sequence. Statistically significant differences are seen when comparing the number of mutations in the core, precore, HBx, and preS1 region of patients with ALF to those without ALF. The comparison between the two groups was performed using the Mann–Whitney *U* Test. SHB = small hepatitis B surface protein, RT = reverse transcriptase, * = *p* < 0.05, ** = *p* < 0.01.

In order to investigate if the degree of variability in the HBV genome of our patient cohort correlated with their clinical course, we compared different viral protein sequences with a genotype-specific reference sequence. As seen in Figure 3, mutations in the preS1 region were significantly more frequent in patients with ALF than in patients without (3(4) vs. 0(3), *p* = 0.017). The same was true for the core region (10(8) vs. 2(4), *p* = 0.009), the precore region (3.5(2) vs. 2(1), *p* = 0.003) and the HBx region (8.5(6) vs. 3(4), *p* = 0.01). We did not observe a statistically significant difference in the number of mutation compared to the genotype-specific sequence for the preS2 region (2(3) vs. 1(3), *p* = 0.173), the SHB region (7(13) vs. 4(7), *p* = 0.128) and the RT region (6(4) vs. 5(6), *p* = 0.363). There was no statistically significant difference in the number of nonsynonymous mutations compared to a genotype-specific reference sequence for the preS1, preS2, SHB, RT, precure, and core regions amongst genotypes A–E. For the HBx region we could detect a significant difference (*p* = 0.006), with genotype D sequences having more differences (6 (6)) compared to the reference sequence than genotype A sequences (2.5 (3)).

### 3.5. Immune Escape Mutations and Stop Codons in ALF Compared to Non-ALF Patients

We evaluated the HBsAg sequences of 8 ALF and 39 non-ALF patients. Immune escape mutations (as presented in the platform Geno2pheno) and stop codon mutations were often found as either majority or minority variants in both groups (see Table 3). We could detect immune escape mutations in 5 (63%) ALF and 21 (54%) non-ALF patients. Overall, the presence of each single mutation was a rare event. Interestingly, a stop codon at amino acid position 216 of HBsAg was found in two ALF patients but not in the non-ALF group (*p* = 0.026). One patient underwent a therapy with methotrexate and infliximab, the other chemotherapy including rituximab. Both patients had genotype A and precore stop codon mutations, high HBV viral loads (1,932,000 and 15,500,000 IU/mL) were treated with entecavir and eventually achieved sustained virological response. One underwent liver transplantation, the other survived without a transplant.

### 3.6. The SHB Stop Codon Mutation at Position 216 Abrogates HBsAg Secretion

**Figure 4:**
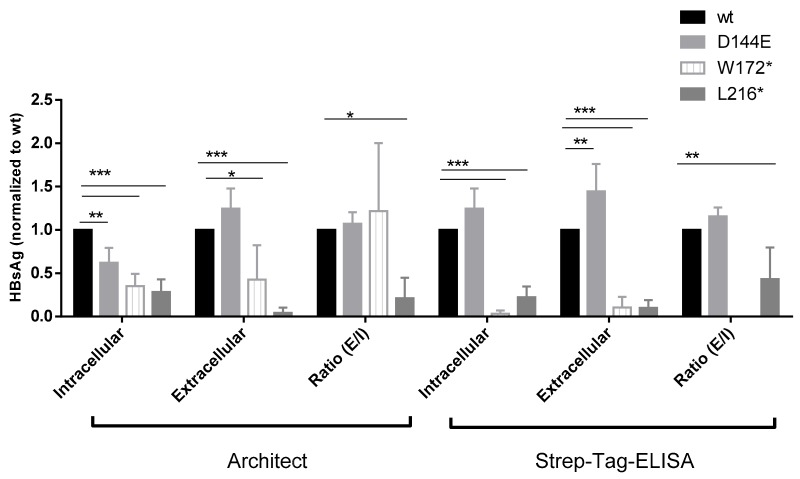
HBsAg production, secretion, and recognition of viral particles harboring HBsAg variants. Huh7 cells were transfected with plasmids coding HBsAg wildtype (wt) or its variants. The HBsAg was measured in cell lysates and supernatant using Architect (Abbott) and Strep-tag ELISA. The HBsAg values for the mutants were normalized to the wt variant. The HBsAg production and secretion (intracellular and extracellular HBsAg) were compared between the wt vs. the SHB D144E, W172* and L216* variant. We detected significantly lower intracellular and extracellular concentrations of HBsAg in the case of the L216* and W172* mutant vs. wt. The extracellular to intracellular ratio of L216* was lower compared to that of the wt. Detection with Architect (Abbott) and Strep-tag-ELISA showed similar results. For the D144E mutant, we observed a modestly reduced intracellular HBsAg (measured with Architect) and a modestly increased extracellular HBsAg (measured with Strep-tag-ELISA) compared to the wt. The ratio of extracellular to intracellular HBsAg of the D144E mutant was comparable to that of the wt. The comparison between the HBsAg of wt and mutants was performed using one-way ANOVA with Dunn’s multiple comparisons test. Wt = wild type, E = extracellular, I = intracellular. * = *p* < 0.05, ** = *p* < 0.01, *** = *p* < 0.001.

Next, we wanted to investigate if the identified mutations that were more frequent in patients with ALF have any influence on the secretion of HBsAg. Therefore, Huh7-cells were transiently transfected with a plasmid encoding the strep-tagged HBsAg, carrying the wild-type (wt) sequence and the SHB L216* mutation, as well as the SHB D144E and W172* as controls (see Materials and Methods section). The impact of the mutations on HBsAg production, secretion, and recognition was evaluated using two different ELISAs (see Materials and Methods section) as depicted in Figure 4. All values are presented as percentages of the wildtype HBsAg. Detection of HBsAg yielded similar results independently from the ELISA that was used (Architect (Abbott) versus Strep-tag-ELISA). We detected significantly lower intracellular (29% ± 15% for Architect (*p* < 0.0001) and 22% ± 12% for Strep-tag-ELISA (*p* < 0.0001)) and extracellular concentrations of HBsAg (4.2% ± 6% for Architect (*p* < 0.0001) and 10% ± 9% for Strep-tag-ELISA (*p* < 0.0001)) in the case of the L216* mutant vs. wildtype HBsAg. The extracellular to intracellular ratio was lower compared to wildtype HBsAg (21% ± 24% for Architect (*p* = 0.049) and 43% ± 36% for Strep-tag-ELISA (*p* = 0.008)). 

A similar pattern was observed for the W172* mutant. We detected significantly lower intracellular (35% ± 14% for Architect (*p* < 0.0001) and 3% ± 4% for Strep-tag-ELISA (*p* < 0.0001)) and extracellular concentrations of HBsAg (43% ± 40% for Architect (*p* = 0.012) and 10% ± 12% for Strep-tag-ELISA (*p* < 0.0001)) in the case of the W172* mutant vs. wildtype HBsAg. The extracellular to intracellular ratio was comparable to wildtype HBsAg (122% ± 79% for Architect (*p* = 0.803) and could not be calculated for Strep-tag-ELISA).

When comparing HBsAg production and secretion of HBsAg D144E mutant compared to HBsAg wildtype, we observed a modestly reduced intracellular HBsAg when measured with Architect (62% ± 17% for Architect (*p* = 0.005) and 125% ± 23% for Strep-tag-ELISA (*p* = 0.054)), and a modestly higher extracellular HBsAg when measured with Strep-tag-ELISA (125% ± 23% for Architect (*p* = 0.347) and 144% ± 32% for Strep-tag-ELISA (*p* = 0.01)). The ratio of extracellular to intracellular HBsAg of the D144E mutant was comparable to wildtype HBsAg (107% ± 13% for Architect (*p* = 0.989) and 116% ± 10% for Strep-tag-ELISA (*p* = 0.594)).

## 4. Discussion

HBV reactivation under immunosuppression is becoming an increasingly important issue, as more and more patients are treated with immunosuppressive regimens. Its clinical manifestation can vary from asymptomatic hepatitis to ALF with fatal outcome [2,4]. The aim of our study was to investigate viral and host factors potentially contributing to ALF in patients with HBV reactivation.

In our cohort, the time point of diagnosis of HBV reactivation was associated with the development of ALF: Patients diagnosed during immunosuppression (diagnosis during chemotherapy and all transplanted patients) had a significantly milder clinical course compared to patients diagnosed at a later time point, after the end of immunosuppressive therapy, as shown in Table 1. This is in accordance with previous observations [5,21] and makes sense from an immunological point of view: HBV is not cytopathic; however, the activation of the immune system during HBV infection, or in our cases the presumed recovery of immunocompetence, can cause considerable liver damage [2]. Another point that supports this hypothesis is the association of anti-HBe presence and ALF in our cohort—the presence of anti-HBe is a sign of partial immune control of HBV infection [1]. This observation is of potential clinical significance—monitoring of these patients is required even after the end of an immunosuppressive treatment since HBV reactivation can occur at this late time point and may have an even worse clinical course than HBV reactivation diagnosed under immunosuppressive treatment.

The clinical and serological background of patients with HBV reactivation was associated with onset of ALF, patients with HBV reactivation due to hematological or oncological malignancies and chemotherapy had an increased risk to develop ALF compared to all other patients. This phenomenon could be partly attributed to the continuous immunosuppression of transplanted patients, which may shield them from fulminant hepatocyte damage. Another potentially synergistic cause could be chemotherapy-induced hepatotoxicity [8,9]. Furthermore, patients with low replicative HBV infection at baseline had an increased risk of developing ALF compared to all other patients. This may be associated with pre-existing liver damage due to HBV in patients with detectable HBsAg at baseline, although in a previous study the percentage of severe/fulminant hepatitis in at baseline HBsAg− and HBsAg+ patients were comparable [22] and in another HBsAg- patients had a greater risk for ALF [4].

Virological parameters such as the HBV and TTV viral load and HBV genotype were not associated with the development of ALF in our cohort. TTV has been proposed as a novel biomarker of the efficacy of immunosuppression in some but not all patient cohorts [6,7]. In a previous study, for example, TTV levels failed to predict immune-related clinical events [23]. In our cohort, the TTV viral load did not differ significantly between patients diagnosed during immunosuppression and patients diagnosed after immunosuppression, nor in patients achieving resolution of the HBV infection compared to patients developing chronic hepatitis B. This may be due to the dynamic nature of immunosuppressive treatment and the fact that TTV viral load was measured only at the time-point of HBV reactivation diagnosis. 

Viral variability has been associated with ALF [12]. In the present study, viral complexity, measured by Shannon entropy, was greater in all viral protein regions (preS1, preS2, SHB, RT, core, precore, and HBx), when comparing HBV sequences from ALF vs. non-ALF patients. In addition, sequences of ALF patients had a greater degree of variability compared to a genotype-specific reference sequence than the sequences of non-ALF patients. This effect could be observed in all protein regions but reached statistical significance in the core, precore, HBx, and preS1 regions. The overwhelming immune response seen in ALF, compared to the possibly more moderate response in non-ALF cases, might apply greater evolutionary pressure on the virus, contributing to increased viral variability and complexity [12]. 

Immune escape mutations in SHB could be often detected in our cohort as either majority or minority variants in accordance with previous studies [4,15]. Amongst the detected immune escape mutations, we found the HBsAg D144E mutation in two ALF and three non-ALF patients. Stop codon mutations were observed in both groups, but more frequently as a majority variant in the ALF group. In particular, the stop codon mutation at amino acid (aa) position 216 of HBsAg was found more frequently in the ALF group. The functional evaluation of L216* demonstrated that this mutation led to reduced production and secretion of HBsAg compared to wildtype HBsAg. This effect was not observed with the D144E mutant. HBsAg stop codon mutations leading to a truncated HBsAg such as W172* have been described in patients with chronic hepatitis B patients in the past and have been associated with lamivudine/entecavir resistance, cirrhosis, and hepatocellular carcinoma, as well as defective HBsAg secretion like L216* [20]. It has been observed that C-terminally truncated middle hepatitis B surface proteins can be retained and accumulated in the endoplasmatic reticulum [20,24]. They may also induce an increased cleavage of procaspases-3 and -9, thus rendering hepatocytes more susceptible to TRAIL-induced apoptosis [25], a pathway that has been implicated in hepatitis-mediated acute liver failure [26,27]. Thus, one could hypothesize that the L216* may contribute to hepatocyte apoptosis and ALF.

Our study has some limitations: It is a retrospective study with varying patient clinical background of the patients and immunosuppressive treatment. Furthermore, although the sample size is comparable or greater compared to previous studies, the statistical power remains limited, when clinical and viral complexity is taken into account. On the other hand, our study has several strengths: Our study addresses an important aspect of HBV reactivation, the risk of development of ALF. We could clearly confirm that not all HBV reactivation episodes are equal in morbidity and mortality. Furthermore, the sequencing of the whole HBV genome per NGS and the functional analysis of the HBsAg L216* mutation allows us to investigate in more depth viral aspects of HBV reactivation-induced ALF.

In conclusion, patients diagnosed with HBV reactivation under immunosuppression had a significantly milder clinical course compared to patients diagnosed after the end of immunosuppressive therapy and immune system reconstitution. Viral sequences from ALF patients showed greater variability compared to sequences from non-ALF patients. Stop codon mutations were observed more frequently as a majority variant in patients with ALF. One of them, the SHB L216* mutation, was associated with reduced HBsAg production and secretion.

## Figures and Tables

**Table 1:** Clinical and laboratory parameters of patients with hepatitis A virus (HBV) reactivation at the time of HBV reactivation diagnosis stratified according to their liver function: acute liver failure (ALF) vs. non-ALF patients.

Variable	ALF *n* = 16 (19%)	Non ALF *n* = 71 (81%)	*p*/Odds Ratio
Sex, M/F (n)	9 (56%)/7 (44%)	46 (65%)/25 (35%)	*p* = 0.573
Age in years	62.5 (17)	57 (20)	*p* = 0.268
BMI (kg/m2), *n* = 76	24.43 (3.99)	24.22 (4.84)	*p* = 0.412
CTx/aHSCT/LTx/NTx	12 (75%)/2 (12.5%)/2 (12.5%)/0 (0%)	29 (41%)/17 (24%)/18 (25%)/7 (10%)	*p* = 0.088
CTx vs. all other causes	12 (75%)/4(25%)	29 (41%)/42 (59%)	***p* = 0.024** ***OR* = 4.34 (1.27–14.81)**
Outcome (resolved infection/ chronic infection/LTx or death/lost on follow up)	4 (25%)/1 (6%)/11 (69%)/0 (0%)	20 (28%)/36 (51%)/9 (13%)/6 (8%)	*p* <0.001
HBV reactivation diagnosis during vs. after immunosuppression	6 (37.5%) vs. 10 (62.5%)	51 (72%) vs. 20 (28%)	***p* = 0.018** ***OR* = 4.25 (1.36–13.24)**
Antiviral therapy with entecavir/tenofovir/other substances/none	11 (69%)/4 (25%)/1 (6%)/0 (0%)	30 (42%)/16 (23%)/8 (11%)/17 (24%)	*p* = 0.106
HBV Reactivation under prophylaxis with entecavir/lamivudine	0 (0%)	3 (4%)/2 (3%)	*p* = 0.579
Presence of hepatic steatosis, *n* = 68	4 (27%)	12 (23%)	*p* = 0.739
INR	2.12 (1.55)	1.02 (0.12)	***p* < 0.001**
Creatinine	0.96 (0.53)	0.95 (0.54)	*p* = 0.738
Bilirubin	11.25 (14.23)	0.7 (0.8)	***p* < 0.001**
AST (IU/L)	1893 (1666)	35 (81)	***p* < 0.001**
ALT (IU/L)	1600 (1451)	43 (134)	***p* < 0.001**

Data are presented as median (IQR) for continuous variables and n, percentage for categorical variables. For comparisons between the groups, we used Chi-Square, Fisher’s exact test, unpaired *t*-Test, or Mann–Whitney *U* test. ALF: acute liver failure, OR: odds ratio, BMI: body mass index, CTx: group of patients with hematological or oncological diseases after chemotherapy, aHSCT: group of patients after allogeneic hematopoietic stem cell transplantation, NTx: group of patients after kidney transplantation, LTx: group of patients after liver transplantation, INR: international normalized ratio, AST: aspartate transaminase, ALT: alanine transaminase.

**Table 2:** Serological and virological parameters of patients with HBV reactivation stratified according to their liver function: ALF vs. non-ALF patients.

Variable	ALF *n* = 16 (19%)	Non ALF *n* = 71 (81%)	*p*/Odds Ratio
Serology before reactivation: HBsAg-/anti-HBc+ vs. HBsAg+/anti-HBc+ vs. anti-HBc+ liver	7 (44%) vs. 7 (44%) vs. 2 (12%)	49 (69%) vs. 6 (9%) vs. 16 (22%)	***p* = 0.002** **1 vs. 2, *p* = 0.003, *OR* = 8.17 (2.12–31.43)** **1 vs. 3, *p* = 1** **2 vs. 3, *p* = 0.017, *OR* = 9.33 (1.5–58.2)**
HBV viral load (IU/mL), *n* = 85	2,821,500 (67,804,900)	1,528,000 (17,999,644)	*p* = 0.192
Anti-HBc IgG, S/CO, *n* = 78	7.74 (3.36)	7.93 (8.13)	*p* = 0.964
Anti-HBc IgM, S/CO, *n* = 72	0.48 (23.08)	0.19 (8.94)	*p* = 0.290
HBsAg, IU/mL, *n* = 71	2605 (3951)	514 (1975)	***p* = 0.012**
HBsAg detection	16 (100%)	56 (79%)	p = 0.063
anti-HBe positivity (n,%) (*n* = 72)	11 (69%)	16 (29%)	***p* = 0.007** ***OR* = 5.5 (1.65–18.36)**
HBV genotype (*n* = 58)	A: 6 (46%)/B: 1 (8%)/C: 0 (0%)/D: 5 (39%)/E: 1 (8%)	A: 19 (42%)/B: 1 (2%)/C: 2 (4%)/D: 20 (44%)/E: 3 (7%)	*p* = 0.811
Concurrent detection of anti-HBs/HBV DNA, *n* = 86	5 (31%)	17 (24%)	*p* = 0.542
HBV DNA detection with undetectable anti-HBc, *n* = 86	1 (6%)	11 (16%)	*p* = 0.450
TTV viral load (copies/mL), *n* = 72	83,000 (5,341,800)	12,800 (455,860)	*p* = 0.136

Data are presented as median (IQR) for continuous variables and *n*, percentage for categorical variables. For comparisons between groups, we used Chi-Square, Fisher’s exact test or Mann–Whitney *U* test. ALF: acute liver failure, OR: odds ratio, TTV: Teno torque virus. S/CO: signal to cut-off ratio.

**Table 3:** Immune escape mutations and stop codons in SHB in patients with HBV reactivation stratified according to their liver function: ALF vs. non-ALF patients.

Variables	ALF *n* = 8	Non ALF *n* = 39	*p*
Presence of immune escape mutations in at least 10% of the sequences, %	4 (50%)	17 (44%)	0.713
Presence of immune escape mutations in at least 1% of the sequences, %	5 (63%)	21 (54%)	0.715
Presence of stop codon mutations in at least 10% of the sequences, %	2 (25%)	0 (0%)	**0.026**
Presence of stop codon mutations in at least 1% of the sequences, %	2 (25%)	6 (15%)	0.609
Immune escape mutations in at least 10% of the sequences per person, n	0.5 (1)	0 (1)	0.989
Immune escape mutations in at least 1% of the sequences per person, n	1 (3)	1 (2)	0.857
Stop codon mutations in at least 10% of the sequences per person, n	1 (2)	0 (1)	0.282
Stop codon mutations in at least 1% of the sequences per person, n	2 (3)	1 (2)	0.588
P105I	0	1	-
L109I/V/R	**1/0/0**	**1** (1)/**1**/2	-
P111 *	0	1	-
G112 *	1	2	-
T118R/K	**0**/1	1/**1**	-
P120A/Q/S/T	**0**/**0**/**0**/1	1/**2**/**2** (1)/**4** (1)	-
R122K	1	**2** (1)	-
T123A	0	1	-
T126S/N/I	**1/0/0**	**0**/**2**/2	-
A128V	**0**	**2**	-
Q129H/R	**0**	**1/3**	-
G130D/N/R	**0**	**1**/**1**/1	-
T131N/I	1/1/0	**1** (2)/1/**1**	-
M133I	0	2	-
F134H/N/S	**0/0/0**	**2/3** (1)/**1**	-
C137Y	**0**	1	-
P142S	**0**	**1**	-
S143L	**0**	**1**	-
D144E/A	**2/0**	**3**/**2** (1)	-
G145R/A	**1/1**	**2**/1	-
W163 *	1	1	-
L172 *	0	1	-
W191 *	0	1	-
W199 *	1	**0**	-
W201 *	1	**0**	-
Y206 *	0	1	-
L216 *	**2**	0	**0.026**
W223 *	0	1	-

Data are presented as median (IQR) for continuous variables and *n*, percentage for categorical variables. For comparisons between groups, we used Chi-Square, Fisher’s exact test or Mann–Whitney *U* test. ALF: acute liver failure, SHB = small hepatitis B surface protein. Individual mutations: Numbers in bold show mutations found in at least 10% of the sequences, other numbers show rarer mutations found in at least 1% of the sequences.
